# Using Virtual Reality to Improve Classroom Behavior in People With Down Syndrome: Within-Subjects Experimental Design

**DOI:** 10.2196/34373

**Published:** 2022-04-07

**Authors:** Stefan Carlo Michalski, Ancret Szpak, Caroline Ellison, Rowena Cornish, Tobias Loetscher

**Affiliations:** 1 UniSA Justice and Society University of South Australia Adelaide Australia; 2 Orana Australia Ltd Adelaide Australia

**Keywords:** virtual reality, Down syndrome, intellectual disability, drawing, art, behavior, mood, attention, classroom, self-report

## Abstract

**Background:**

People with Down syndrome face various learning challenges. Introducing new and enjoyable experiences in learning settings may improve learning outcomes. Immersive and interactive technologies such as virtual reality can be used to deliver rich visual experiences in classrooms.

**Objective:**

The aim of this study was to investigate the feasibility and benefits of virtual reality exposure for people with Down syndrome in learning settings.

**Methods:**

To address this aim, we used a within-subjects design to assess the effect of a brief virtual reality drawing experience and conventional drawing experience on subsequent behavior in 16 participants.

**Results:**

Large positive effects were found for virtual reality drawing (*t*_15_=5.020, *P*<.001) and conventional drawing (*t*_15_=3.720, *P*=.002) in improving subsequent behavior in a learning setting. Irrespective of the intervention, the participant’s mood, attention, and overall behavior significantly improved. No significant differences were found between the interventions (*t*_15_*=–*0.648; *P*=.53).

**Conclusions:**

This study’s results are encouraging for researchers and educators interested in using virtual reality for people with Down syndrome, as virtual reality was found to be highly feasible. Recommendations are made for researchers and educators interested in providing virtual reality experiences for people with Down syndrome.

## Introduction

People with Down syndrome often encounter significant challenges in learning settings [1]. Inattentiveness, impulsive behavior, excessive fidgeting, and other nondirected motor activity are signs of distress and stereotypical behaviors for people with Down syndrome [2]. Immersive technologies such as virtual reality (VR) hold great potential in delivering enjoyable and therapeutic experiences [3]. VR is commonly being considered by researchers and educators to provide safe access to realistic experiences that may otherwise be logistically difficult, dangerous, or impractical to implement [4,5]. Exposure to VR settings that provide a sense of distance from routine can reduce stress and improve mood [6]. There is encouraging evidence supporting the use of nonimmersive virtual environments to provide useful learning [7], rehabilitation [8], and leisure experiences for people with intellectual disabilities. Therefore, immersive VR applications may also be an effective way to improve motivation and engagement for people with Down syndrome in learning settings.

Applications of VR are proliferating in psychology, health care, and education [9]. Weiss et al [10] and Yalon-Chamovitz and Weiss [11] were few of the first researchers to study the use of VR to improve the leisure experiences of people with intellectual disabilities. In these experiments, virtual environments were found to increase enjoyment and engage participants with cerebral palsy and an intellectual disability [10,11]. Flat-screen displays were used to deliver the experience, offering a low level of immersion. Given these positive early results in people with intellectual disabilities, it is surprising that there is limited research investigating the use of modern and more immersive virtual environments such as head-mounted displays (HMDs). Modern technology offers new opportunities, and there is reason to believe that more specific, realistic, and engaging VR applications may be useful for people with intellectual disabilities.

HMDs may be preferred over less-immersive displays as it can provide rich visual experiences that elicit greater feelings of presence in the user [12]. However, implementing immersive technology in vulnerable or VR-inexperienced groups needs to be carefully introduced and monitored for possible negative experiences specific to that population. People with Down syndrome, for example, have structural eye abnormalities, which may diminish their vision even when corrected [13]. There is a high prevalence of near vision impairments in people with Down syndrome, and 25%-60% have strabismus [13], which will affect their visual perception when using stereoscopic displays. A convergence insufficiency (inability to maintain binocular function) will likely lead to difficulty seeing depth in VR, which may also increase the likelihood of a negative VR experience [14,15].

People with Down syndrome may be predisposed to experience cybersickness. Cybersickness has been related to a visual-vestibular mismatch between VR and the real world, leading to symptoms such as nausea or disorientation [16]. Vergence-accommodation conflicts may also exacerbate oculomotor symptoms such as eye strain and fatigue [14,17]. It is unclear how enjoyable VR is for people with eye abnormalities, given many typical users still experience adverse effects. In addition, people with Down syndrome typically have difficulties with fine motor skills due to low muscle tone and joint hypermobility [18], which may present challenges when interacting in virtual environments. Thus, it is unclear how suitable using a headset and a handheld controller is. A thorough investigation of users’ experiences with HMDs is critical.

Self-report measures are typically used to assess VR aftereffects, though such measures must be interpreted with caution in this population. Widely reported in the literature are concerns that people with intellectual disabilities tend to positively self-report or overestimate their responses [19,20]. For example, Yalon-Chamovitz and Weiss [11] found in their study on young adults with cerebral palsy and moderate intellectual disability that self-reported success and enjoyment in VR significantly differed from staff observations. Researchers and educators alike encounter significant challenges in obtaining valid self-reports from participants with intellectual disabilities owing to challenges in communication and comprehension, especially among nonverbal participants [21]. Utilizing methods that do not use complex language, such as observation, may prove to be more effective when assessing behavior in learning settings.

The aim of this study was to investigate the feasibility and benefits of VR exposure for people with Down syndrome. To address this aim, we explored the effect of a brief VR drawing experience and conventional drawing experience on subsequent behavior in a learning setting. Drawing was selected as it is a familiar activity, and participants could engage in free play.

## Methods

### Design

This study contrasted 2 drawing activities in a within-subjects design: drawing in a VR application (Tilt Brush, developed by Google) and conventional drawing. The researcher removed the participants from the learning setting to complete each activity. Once completed, the participants returned to the learning setting for observation. Participants were required to wait a minimum of 24 hours before completing their second activity (counterbalanced order).

### Participants

Seventeen people (mean age 25.25 [SD 6.61] years) diagnosed with Down syndrome were recruited from a nonprofit disability services organization in South Australia. One participant was excluded as they were unable to complete the VR experience. Thus, 16 participants were included in the analyses (7 females and 9 males). All participants in this study were considered to have a severe-to-profound intellectual disability. The severity of the intellectual disability was classified on the ability to perform daily skills as per the Diagnostic and Statistical Manual of Mental Disorders, fifth edition criteria [22]. All participants attended a program that aimed to improve life skills in young adults with Down syndrome. Class sizes varied, though the number of clients never exceeded 12 per session. At a minimum, 1 support staff member was present per 4 clients. Informed consent was obtained from the participant, caregiver, and a staff member at the organization. An easy-to-read consent form with pictures was developed to ensure that participants clearly understood what was involved in the study. The appropriate sample size was calculated using the G*Power 3 software [23]. Yalon-Chamovitz and Weiss [11] found a large effect size for perceived level of enjoyment in a VR leisure activity in people with physical and intellectual disabilities. Using a large effect size (0.80) as an estimate for the power analysis, it was calculated that for 1-sample 1-tailed *t* tests, at least 15 participants would be needed to suffice power with α=.05.

### Ethics Approval

This study was granted ethics approval from the University of South Australia Human Research Ethics Committee (202640).

### Materials

#### VR Apparatus

The Oculus Quest (developed by Facebook Technologies, LLC) was used. Immersive HMDs such as the Oculus Quest enable users to view a 3D environment that moves in real time following their movements. Users were required to hold a controller to interact within the environment while wearing the HMD. Corrective glasses were worn in the device if needed.

#### VR Application

Tilt Brush (developed by Google) was used. Users were immersed in a 360° virtual environment where they could paint and observe their artwork in 3D space. A controller was used to simulate a paintbrush in the virtual environment.

#### Conventional Drawing

Participants were provided a blank A4 paper and their favorite color pencil.

### Measures

#### Learner Behavior

Learner behavior data were collected from support staff at the disability services organization. Staff observed participants and provided ratings for changes on the following 6 factors (proceeding examples were also listed): (1) mood, (2) attention (eg, listening to instructions, not distracted, not looking around), (3) activity (eg, jumping out of the seat, walking around class inappropriately), (4) impulses (eg, blurting out answers before questions completed, interrupting others, butt into conversations, failing to wait turn), (5) anxiety (eg, fidgeting, bite hands/nails, twitch, pace, shake, hand/feet tapping, tense expression), and (6) withdrawal (eg, staring blankly, daydream, fiddling with objects, detached). Fifteen minutes after returning to the learning setting, 2 staff rated changes in behavior on a 7-point scale ranging from better (+3) to worse (–3), with 0 being no change. The 2 rater scores were averaged. Individual subscales and the total scores (sum of all subscales) were analyzed. The staff remained blinded to which intervention the participant completed (ie, VR drawing or conventional drawing). Further, staff were asked to report any noticeable observations in the participant’s behavior (ie, reports of sickness or suspicion of the activity completed). The learner behavior form was adapted from Part 2 of Mather and Jaffe’s [24] classroom behavior form, which was designed to observe problem behaviors in a classroom. This measure demonstrated good internal consistency in this study (Cronbach α=.72). The learner behavior form used in this study is available upon request.

#### Drawing Time

The number of minutes participants opted to remain in each drawing activity was recorded.

#### Cybersickness

The researcher asked participants if they were feeling dizzy or sick. Specifically, the researcher handed the participants a sheet that stated, “I felt dizzy or sick…” Below this statement, there were 3 response options: no, not sure, or yes. Each option had an emoticon underneath, a smiley face, confused face, and a nauseous face, respectively. Participants were required to select an option by either circling the response on paper or by saying the word aloud. The researcher guided the participants through the question to ensure comprehension.

#### Choice Paradigm

The researcher asked questions regarding preference of 3 different activities: drawing in VR, drawing on paper, and watching TV. Questions were phrased in 3 different ways:

1. Single-choice question: participants provided a yes or no response to each activity individually, indicating whether they enjoy the activity and would like to do it again in the future. The researcher asked, for example, “Do you like drawing in virtual reality?”
2. Paired-choice question: participants were asked to select their favorite activity out of 2 options. The researcher asked, for example, “What do you like better: drawing in virtual reality or drawing on paper?” The paired choice was completed when participants responded to each of the 3 pair combinations. Based on the responses, their favorite item was determined.
3. Multiple-choice question: participants were asked to select their overall favorite out of the 3 activities. Specifically, the researcher asked, “What is your favorite activity out of drawing in virtual reality, drawing on paper, or watching TV?”

### Procedure

In a counterbalanced order, participants completed 2 interventions: VR drawing and conventional drawing. Participants were not given specific instructions on what to draw, as the researcher indicated they had free time. The range of options in the VR drawing was replicated in the conventional drawing as best as possible. For example, in-game sounds and effects were removed. Furthermore, in both conditions, the researcher asked participants for their favorite color and that was the only color used. Participants held only 1 controller and 1 pencil in each experience. Similarly, participants did not have erasers, and both conditions were completed on a neutral background.

Participants were instructed that 7 minutes had been allocated to each activity. Once 7 minutes elapsed, the researcher asked the participants if they would like to continue for an additional minute or stop entirely. If participants opted to continue, this process was repeated at the end of each minute until a maximum of 10 minutes was reached. Although participants were informed that 7 minutes had been allocated to the activity, they were reminded they could withdraw at any time. After both interventions, the researcher asked the participants if they were feeling dizzy or sick and recorded notes.

The choice paradigm was completed following the second intervention, where the researcher asked the single-choice, paired-choice, and multiple-choice questions. Physical props were used (ie, VR headset, paper and pencils, and an image of a TV) to ensure participants understood the questions.

Following completion of each activity, learner behavior data were collected. Two assigned staff members were asked to observe the participant for 15 minutes upon returning to the learning setting. Then, the staff members were asked to complete the learner behavior form by providing independent ratings based on their observations. Staff were asked to note if they had a suspicion what activity the participant completed. Staff carried on with the regular class routine, which means they may have worked with multiple clients simultaneously.

## Results

### Feasibility

Twenty-two people were invited to participate, but only 17 agreed. VR exposure was highly feasible in this sample, with 16 out of 17 participants able to complete the VR activity. One participant was excluded as they did not engage in the task and were nonresponsive; therefore, the researcher opted to discontinue the experience. Two out of 16 participants (13%) elected to end the VR experience before the allocated time expired. Five out of 16 participants (31%) reported cybersickness symptoms after VR exposure. The researcher followed up on the participants’ symptoms: 3 reported eye strain, 1 reported dizziness, and 1 was unable to provide further information. All symptoms reported were mild and short-lived, as there was no evidence of discomfort during observations (15 minutes after the exposure). A paired-sample *t* test revealed no difference of drawing time between VR drawing (mean 8.0 [SD 2.28] minutes) and conventional drawing (mean 7.5 [SD 2.03] minutes; *t*_15_=0.6; *P*=.54; Cohen *d*=0.16).

### Learner Behavior

A series of paired-samples *t* tests were conducted to assess whether there were significant differences in learner behavior following the VR drawing and conventional drawing interventions. Paired-samples *t* tests revealed no significant differences between the 2 interventions (Table 1).

A series of 1-sample *t* tests were conducted to assess whether the interventions changed behavior from 0 (representing no change, Table 2). One-sample *t* tests revealed that the total score of learner behavior was significantly different from 0 for VR drawing (*P*<.001) and conventional drawing (*P*=.002) interventions. Mood and attention scores were also significantly different from 0 after both interventions. Notably, the effect sizes were large following the VR intervention and bigger in comparison to that following conventional drawing. Furthermore, activity scores increased after VR, while withdrawal scores increased after conventional drawing. Nonsignificant differences were found in the remaining variables.

**Table 1:** Learner behavior differences between virtual reality drawing and conventional drawing interventions.

Variable	Mean (SD)	*t* value *(df)*^a^	*P* value	Cohen *d*^b^
Mood	0.61 (0.5)	–0.131 (15)	.89	0.03
Attention	0.66 (0.4)	–0.324 (15)	.75	0.08
Activity	0.17 (0.2)	0.863 (15)	.40	0.21
Impulses	0.22 (0.3)	–0.307 (15)	.76	0.07
Anxiety	0.19 (0.3)	–1.103 (15)	.29	0.27
Withdrawal	0.23 (0.3)	–1.454 (15)	.17	0.36
Total^c^	2.08 (1.4)	–0.648 (15)	.53	0.16

^a^Instances of negative *t* values indicate higher scores in conventional drawing as compared to those in virtual reality drawing.

^b^Cohen *d* effect size interpretation: 0.2=small effect size, 0.5=medium effect size, and 0.8=large effect size.

^c^Total score indicates the sum of all subscales.

**Table 2:** Learner behavior differences for each variable in each intervention.

Variable, intervention		Mean (SD)^a^	*t* value *(df)*	*P* value	Cohen *d*^b^
**Mood**					
	Virtual reality drawing	0.59 (0.5)	4.842 (15)	*<.001* ^c^	1.21
	Conventional drawing	0.63 (0.8)	2.953 (15)	*.01* ^c^	0.74
**Attention**					
	Virtual reality drawing	0.63 (0.5)	5.371 (15)	*<.001* ^c^	1.34
	Conventional drawing	0.69 (0.6)	4.198 (15)	*<.001* ^c^	1.05
**Activity**					
	Virtual reality drawing	0.25 (0.4)	2.449 (15)	*.03* ^c^	0.61
	Conventional drawing	0.09 (0.4)	0.899 (15)	.38	0.22
**Impulses**					
	Virtual reality drawing	0.19 (0.4)	1.861 (15)	.08	0.46
	Conventional drawing	0.25 (0.6)	1.732 (15)	.10	0.43
**Anxiety**					
	Virtual reality drawing	0.09 (0.4)	1.000 (15)	.33	0.25
	Conventional drawing	0.28 (0.5)	2.058 (15)	.06	0.51
**Withdrawal**					
	Virtual reality drawing	0.09 (0.3)	1.379 (15)	.19	0.34
	Conventional drawing	0.38 (0.6)	2.423 (15)	*.03* ^c^	0.61
**Total^d^**					
	Virtual reality drawing	1.84 (1.5)	5.020 (15)	*<.001* ^c^	1.25
	Conventional drawing	2.31 (2.5)	3.720 (15)	*.002* ^c^	0.93

^a^A mean score of 0 represents no change. Higher scores reflect better behavior. If the blinded staff members guessed the activity a participant completed, they were correct only at chance level (17/32, 53%).

^b^Cohen *d* effect size interpretation: 0.2=small effect size, 0.5=medium effect size, and 0.8=large effect size.

^c^Significant values (*P*<.05) are italicized.

^d^Total score indicates the sum of all subscales.

### Self-reported Activity Preference

**Figure 1:**
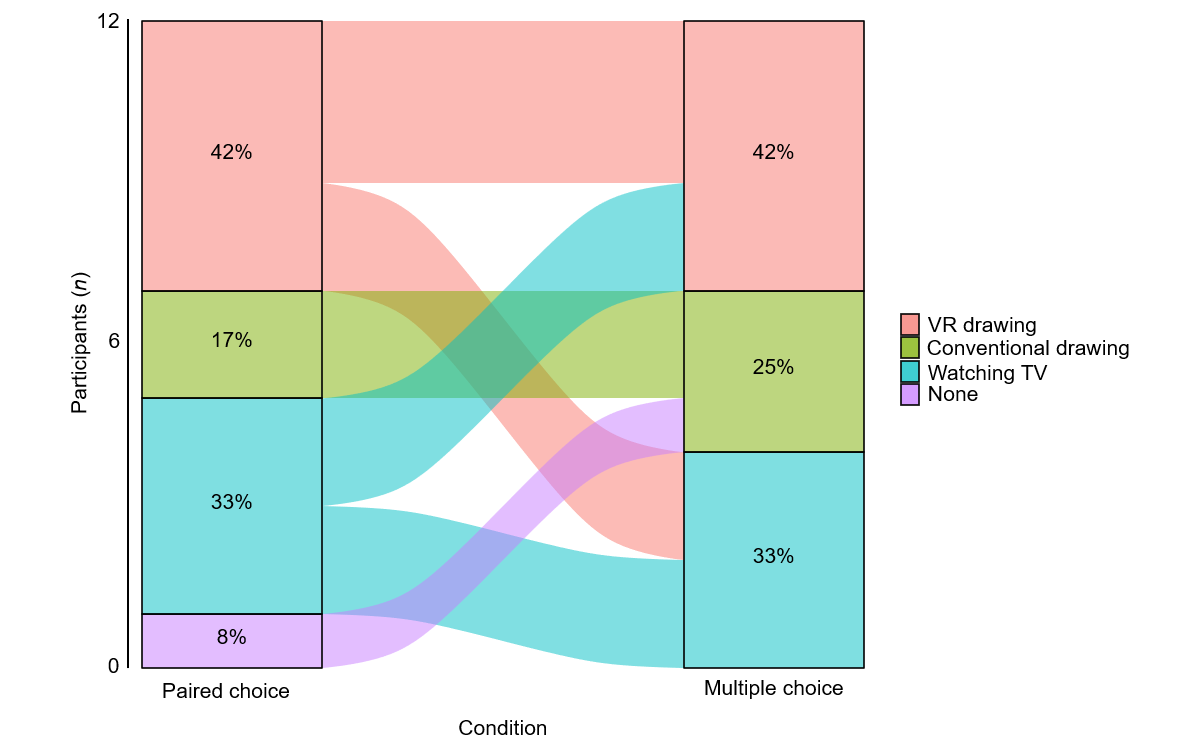
Alluvial plot showing self-reported activity preference for virtual reality drawing, conventional drawing, and watching TV under different formats (paired choice versus multiple choice). The alluvial plot highlights the percentage of participants who had an inconsistent response when asked about the same topic (activity preference) in a different way. VR: virtual reality.

Twelve participants responded to the choice paradigm. The remaining 4 participants were unable to complete the choice paradigm as they were nonresponsive to the questions. Regarding single stimulus responses, all participants provided a yes response for both VR and conventional drawing, indicating their enjoyment of each activity. Figure 1 shows the percentage of participants’ responses in paired and multiple-choice responses. Five out of 12 participants (42%) who completed the choice paradigm were inconsistent upon a comparison of their paired choice and multiple-choice responses. The alluvial plot highlights the inconsistency in the participant’s self-report when asking questions in different formats.

## Discussion

Overall, learner behavior was found to improve after both VR drawing and conventional drawing in people with Down syndrome and a severe-to-profound intellectual disability. As assessed by blinded staff members, there was no evidence that one intervention was more effective than the other. Perhaps participants found the break from the learning setting most valuable, irrespective of the activity. Although the total score of learner behavior significantly improved, not all factors improved. After both activities, considerable improvements were found for mood and attention, while the differences for impulses and anxiety were nonremarkable. Impulsiveness is rigid and perhaps less likely to change from a brief intervention. Attention deficits in people with Down syndrome are well documented, though effective ways to improve them are less understood. The findings from this study are encouraging, as 10 minutes or less of conventional and VR drawing improved learner behavior, which may subsequently improve learning success.

It stands to reason that there is scope to amplify the observed positive effects. First, drawing is likely not the preferred activity for all participants. An advantage of VR is to easily tailor an experience or activity to the specific preferences of a user. Perhaps being more selective with the activities could have improved engagement and the subsequent effects on behavior. Second, some participants may have preferred clearly structured activities as opposed to free play as this is more common at the organization. Third, the researcher removed participants from the learning setting at any time to start the activity. It may have been more effective to conduct the intervention during instances when participants were exhibiting poor or undesirable learning behavior. Consideration of these factors is an important direction for further study.

The key aim of this study was to assess the feasibility aspects of using VR in people with Down syndrome. Of the 22 people invited to participate, 17 agreed, indicating the willingness of the participants and their guardians to participate in research using VR technology. Sixteen out of 17 participants were able to engage in the VR activity successfully. The findings from this study demonstrate the feasibility of VR use in young adults with Down syndrome and severe-to-profound intellectual disability. Overall, VR was tolerated well. Of the 16 participants, 2 (13%) elected to end the VR experience before the allocated time expired, and 5 (31%) reported visual discomfort symptoms after VR, including eye strain and dizziness.

Given that participants’ verbal abilities were limited, they were not able to clearly quantify the severity of their symptoms. Behavioral observations were therefore essential to detect if participants appeared distressed after exposure to the intervention. During these observations, no staff member reported unusual behavior or other significant concerns. Furthermore, behavior in the learning setting improved. We take this as good evidence that there were no serious negative effects of VR exposure. If many participants were sick, we would expect to see a negative impact on behavior. Yet, we found the opposite. The improved ratings and lack of distress identified in behavioral observations indicate acceptable levels of cybersickness in this study, as all symptoms were mild and short-lived. It is important to note that participants may have presented with greater positive behavior despite negative aftereffects owing to a novelty effect, as this was perhaps their first time using VR technology. It remains an open question to what degree a novelty effect contributed to the results.

Although there were no obvious major concerns of cybersickness, it is highly recommended that the interpupillary distance (IPD) be measured and adjusted in headsets to reduce the likelihood of visual discomfort. IPD is the distance between the pupils of both eyes, which facilitates the correct positioning of VR headset lenses. IPD range is essential for optimal image quality, comfort, and has been related to cybersickness in HMDs [25]. In VR, specific points on the lenses must coincide with the center of each eye’s pupil (visual axis) for the display image to be in focus [25]. In our study, participants were unable to adjust the IPD in VR. If a VR headset does not allow such eye lens alignment, eyestrain and headaches can be expected [25]. It is plausible that the user’s inability to adjust IPD in this study contributed to the experiences of cybersickness. 

There was a large variability found between participants’ abilities in understanding instructions when using the device. In this study, participants engaged in a simple activity by using basic functions on a handheld controller. The authors note that basic functionality in the application was restricted as 2 controllers are typically used to select in-game options. It is unclear if the success found in this study would translate to VR tasks that require more complex interactions, as it was found that many participants needed assistance before engaging in the task independently.

The choice paradigm was designed to assess self-reported activity preference; yet, this measure’s findings highlight a more significant issue. It was found that 5 out of 12 participants (42%) provided inconsistent responses when selecting their preference. For example, a participant may have selected activity A as preferred over activities B and C during the paired-choice options, but when asked to select their favorite overall, they selected B. This measure highlights the difficulty of obtaining valid self-report measures in people with Down syndrome [19]. Objective measures are critical for assessing the safety of VR for this population. Standardized measures such as the simulator sickness questionnaire [26] are typically used to capture cybersickness. Stereoacuity measures would also help measure stereovision (the ability to perceive depth), which is another essential component for an enjoyable VR experience. However, there are no suitable and standardized measures for this group. Despite the challenges with self-report in this population, it is important that measures of cybersickness and stereoacuity are not neglected. Valid assessments are needed that accommodate the language barriers in this sample.

This study’s results are encouraging, as VR usage was found to be highly feasible for people with Down syndrome. Participants enjoyed the VR experience and engaged in the task well. There were some experiences of cybersickness; however, all were mild and short-lived. Large positive effects were found for a brief drawing experience to improve overall learner behavior following both VR and conventional interventions. This suggests that immersive VR exposure may provide similar benefits to traditional (paper and pencil) options. For researchers and educators interested in using VR in similar samples, it is recommended that measures are carefully operationalized and there is limited reliance on self-report. Assessing cybersickness is essential to ensuring that users engage in a positive VR experience. An observation checklist worked well in this study to determine the frequency of positive and negative behaviors after exposure to the VR intervention. In this experiment, VR was used as a tool for leisure. Based on the success identified in this study, researchers could investigate the potential for people with Down syndrome to complete tasks and develop real-world skills via training in VR. VR provides promise as a tool to practice real-world skills in a safe, repeatable, and controlled environment [27], though further research is required.

## Abbreviations

HMD: head-mounted display
IPD: interpupillary distance
VR: virtual reality
